# Giant Desmoid Fibromatosis of the Small Bowel After Sleeve Gastrectomy: Case Report and Review of the Literature

**DOI:** 10.3390/jpm16080402

**Published:** 2026-07-27

**Authors:** Teresa Sinicropi, Carmelo Mazzeo, Mariausilia Franchina, Maria Iannello, Francesco Fleres

**Affiliations:** 1 Department of Human Pathology of the Adult and Evolutive Age “Gaetano Barresi”, Section of General Surgery, University of Messina, Via Consolare Valeria, 98125 Messina, Italy; carmazzeo@unime.it (C.M.); maria.iannello.md@gmail.com (M.I.); 2 Department of Human Pathology of the Adult and Evolutive Age “Gaetano Barresi”, Section of Pathology, University of Messina, Via Consolare Valeria, 98125 Messina, Italy; mariausiliafranchina@gmail.com

**Keywords:** desmoid tumor, mesenteric tumor, mesenteric fibromatosis, desmoid fibromatosis, bariatric surgery, sleeve gastrectomy, personalized medicine, individualized management

## Abstract

**Introduction:** Desmoid fibromatosis (DF) is a rare, locally invasive, and typically non-metastatic neoplasm. Its pathophysiology is poorly understood, and the subtle clinical presentation makes diagnosis challenging. A multidisciplinary approach is necessary to achieve a definitive diagnosis and identify the most effective therapeutic strategy. Because of its heterogeneous presentation and unpredictable behavior, DF cannot be managed through a standardized protocol, and diagnostic and therapeutic decisions must instead be tailored to the individual patient, consistent with a personalized-medicine approach. We present an unusual case in which we aim to evaluate the diagnostic procedures and treatments used and determine whether they align with the recent literature. **Materials and Methods**: A case report. A 27-year-old man presented with fever and worsening abdominal pain, without clinical signs of intestinal obstruction. He had undergone a sleeve gastrectomy five years earlier. A computed tomography (CT) scan revealed an intra-abdominal mass in the mesohypogastric region, likely originating from the mesentery of the small intestine. The patient underwent a right hemicolectomy extended to the terminal ileum. Histopathological examination and immunohistochemistry confirmed the diagnosis of DF. The patient was discharged on the 5th postoperative day (POD) in good general clinical condition. To date, there are no signs of recurrence. **Conclusions**: The multidisciplinary approach is essential for managing DF and R0 surgical resection remains the gold standard. The diagnostic and therapeutic pathway followed in our patient appears consistent with the recent literature. This case illustrates how individualized clinical reasoning can be applied even to a rare, heterogeneous neoplasm for which no standardized protocol exists. Furthermore, an established post-surgical management protocol for DF has not yet been developed. Additionally, we observed a possible, hypothesis-generating association between bariatric surgery and the subsequent onset of an intra-abdominal desmoid tumor; given the rarity of desmoid fibromatosis relative to the high volume of bariatric procedures performed worldwide, this observation should not be interpreted as a confirmed correlation, and we conducted a literature review to explore it as a hypothesis. Numerous studies are needed on this matter, but we hope that this case can provide a small starting point for subsequent studies.

## 1. Introduction

Desmoid fibromatosis (DF), also known as desmoid tumor (DT) or aggressive fibromatosis, is a rare soft tissue neoplasm [1]. It accounts for approximately 0.03% of all neoplasms and less than 3% of soft tissue tumors. Its incidence is 2 to 4 cases per million people, with a predominance in women, who are affected at twice the rate of men [2].

The World Health Organization (WHO) classification of soft tissue tumors defines DF as an intermediate, locally aggressive neoplasm [3]. Despite its histologically benign nature and lack of metastatic potential, DF demonstrates a significant propensity for local invasion and recurrence following surgical resection [4].

DTs are categorized based on their etiology and anatomical location, such as the abdominal wall, intra-abdominal or extra-abdominal [5]. Intra-abdominal fibromatosis, also known as primary mesenteric fibromatosis [6], accounts for only 10–20% of all DTs [2,7].

Importantly, desmoid tumors are a well-recognized manifestation of FAP: approximately 10–30% of DTs arise in the genetic, Gardner-syndrome/FAP-associated setting [8] (discussed further below), and conversely, a substantial proportion of patients with FAP develop a desmoid tumor during their lifetime, most often following abdominal surgery such as colectomy. Because DF, and particularly intra-abdominal DF, can therefore be a sentinel manifestation of FAP, a diagnosis of DF should prompt consideration of an underlying polyposis syndrome, and a diagnostic colonoscopy is advisable to exclude colorectal polyposis.

Imaging and histopathological findings are crucial for the differential diagnosis, especially when ruling out lymphoma, soft tissue sarcomas, and gastrointestinal stromal tumors (GISTs).

Given this heterogeneity in presentation, location, and behavior, DF cannot be approached through a single standardized protocol; diagnostic work-up and treatment must instead be individualized to each patient’s anatomy, risk profile, and clinical context, in line with a personalized-medicine paradigm. We report the case of a young male patient with intra-abdominal DF, who initially presented with acute abdominal pain, fever, and diarrhea, to illustrate how such an individualized diagnostic and therapeutic pathway was applied in practice.

## 2. Materials and Methods: Case Report

A 27-year-old man presented to the emergency department with increasing lower abdominal pain, fever, and diarrhea lasting approximately 24 h. He reported fatigue and headache persisting for 1 month.

The patient’s surgical history included a sleeve gastrectomy performed 5 years earlier and a tonsillectomy. He had no history of drug abuse or allergies, and there was no significant family medical history.

Regarding the patient’s metabolic profile, his body mass index (BMI) prior to sleeve gastrectomy was (37.8) kg/m^2^, decreasing to (29.9) kg/m^2^ at the time of the desmoid tumor resection, five years after bariatric surgery. He also had insulin resistance after bariatric surgery (fasting glucose at admission: 146 mg/dL; HbA1c: not available; HOMA-IR: not available). No dedicated endocrine work-up (e.g., testosterone, gonadotropins, thyroid function beyond routine screening) was performed as part of the diagnostic pathway for the desmoid tumor.

On admission his vital signs were as follows: temperature of 37.8 °C, blood pressure of 120/80 mmHg, heart rate of 110 beats per minute, and respiratory rate of 16 breaths per minute. Laboratory findings were within normal limits. Abdominal examination revealed a firm, nontender mass in the midline.

**Figure 1:**
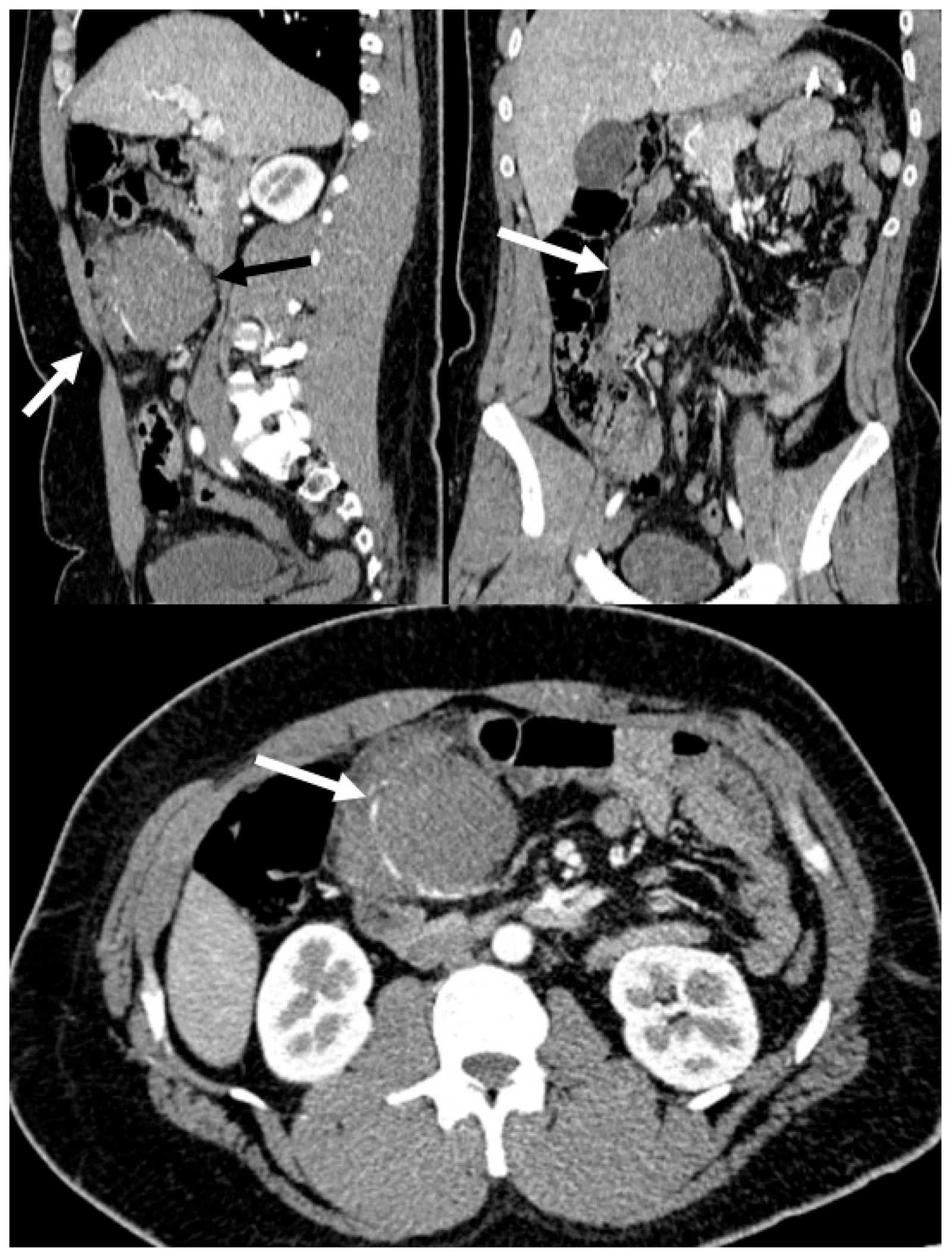
The CT scan reveals the abdominal mass (white arrows) compressing the vena cava (black arrow).

CT with intravenous contrast showed an intra-abdominal mass in the mesohypogastric region. The mass appeared to arise from the mesentery and seemed inseparable from adjacent ileal loops. Its maximum dimensions were approximately 94 mm × 73 mm × 90 mm. It notably displaced branches of the right colic artery and showed moderate contrast enhancement. Multiple lymphadenopathies were also present, each measuring up to 15 mm, with no evidence of lesions involving the lungs and intra-abdominal organs (Figure 1).

**Figure 2:**
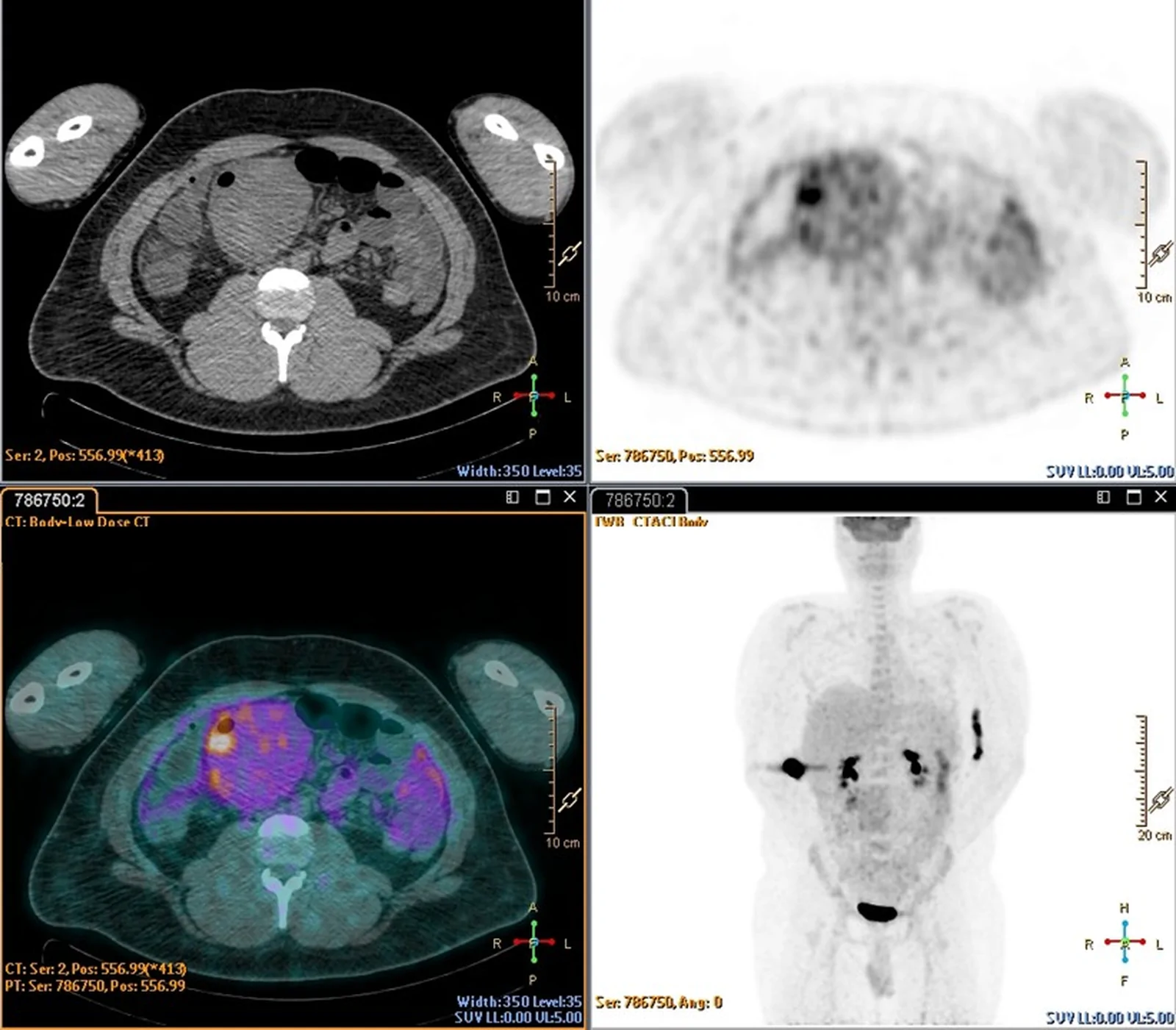
PET showing modest and inhomogeneous uptake of the glucose metabolism tracer corresponding to a known pathological mass located in the meso-hypogastric region. No significant focal hyperaccumulation is observed. However, multiple mesenteric lymphadenopathies are present. Additionally, low and inhomogeneous metabolic activity is noted within the pelvic cavity, likely related to an abundant fluid component that appears inseparable from the intestinal loops.

Positron emission tomography (PET) demonstrated modest and irregular uptake of the glucose metabolism tracer at the known mass (Figure 2).

Tumor markers were CEA 0.85 ng/mL; AFP 1.20 ng/mL; CA 19-9 2.29 [IU]/mL; CA 125 2.68 U/mL; CA 15-3 14.70 [IU]/mL; CA 50 1.70 U/mL.

The operative report of the initial bariatric surgery noted no peritoneal adhesions, abnormal anatomo-pathological findings, or deviations in trocar positioning compared to a standard laparoscopic sleeve gastrectomy.

The patient underwent an exploratory laparotomy. Intraoperatively, the mass appeared to originate from the intestinal mesentery and was compressing the terminal ileal loop. Exploration revealed diffuse serous ascites and a jejuno-ileal bowel obstruction caused by the previously noted bulky exophytic mass. This mass was infiltrating the corresponding ileal mesentery and the terminal ileal loops (approximately 40 cm from the ileocecal valve).

The mass also exhibited a suspicious contact area suggestive of infiltration into a segment of the sigmoid colon on its antimesenteric border, and loose adhesions were present between the root of the jejuno-ileal mesentery and the gastric resection margin.

**Figure 3:**
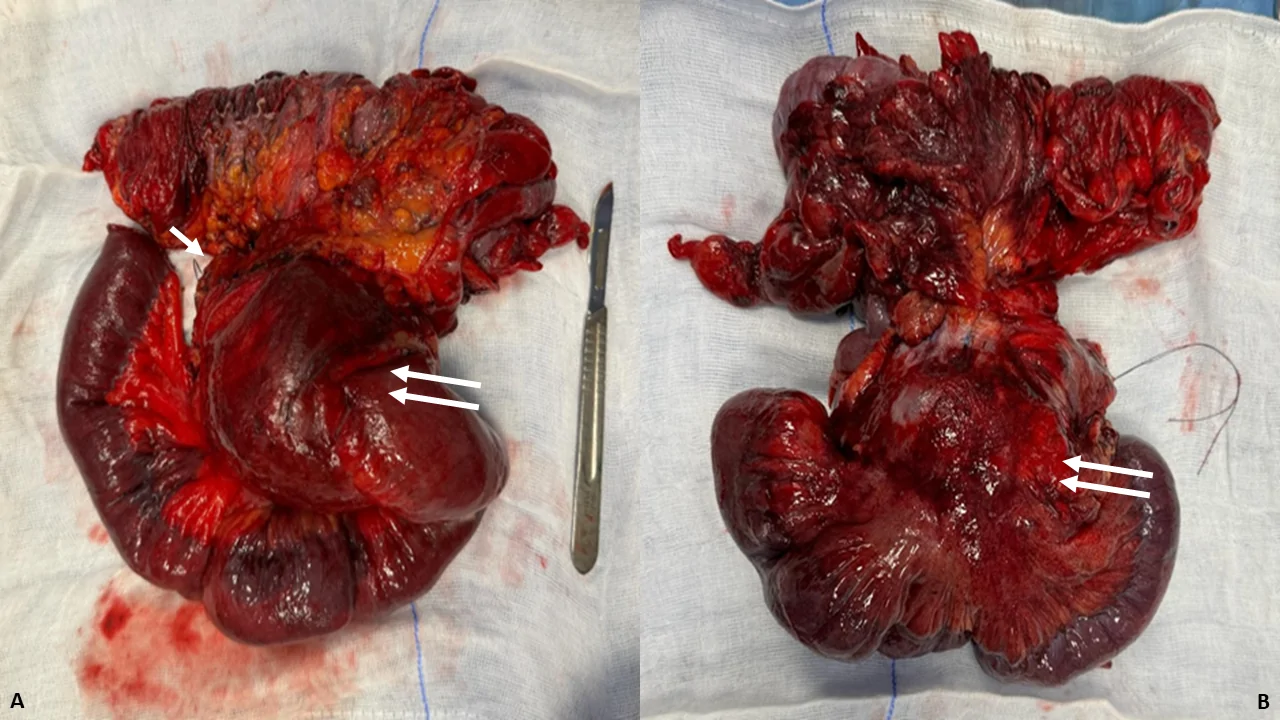
Intraoperative specimen with en-bloc resection of the mass. In (**A**), the white arrow shows ligation of a distal segmental branch of the superior mesenteric vein involved by the mass (the ligature can be seen)—the main trunk of the superior mesenteric vein was preserved throughout the procedure to avoid bowel ischemia. The double arrow demonstrates the encasement of the intestinal loops and mesenteric retraction. In (**B**), the double white arrow shows the posterior portion of the mesentery, which is retracted and involved by disease.

**Figure 4:**
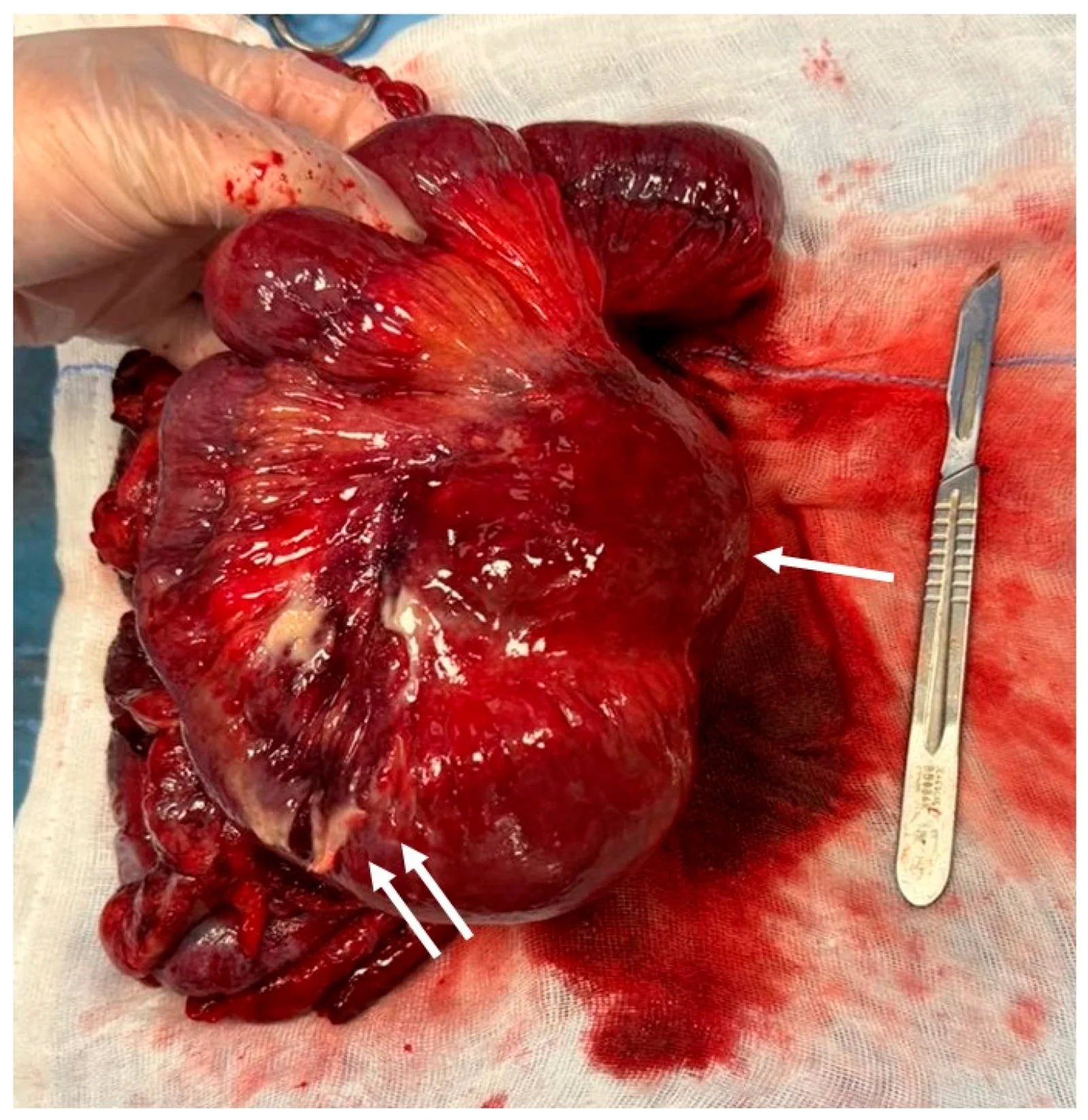
Intraoperative specimen with en-bloc resection of the mass, with initial necrosis of the jejunal mesentery and the jejunal loop (double arrows). The white arrow shows the root of the mesentery.

An en-bloc resection of the tumor was performed, including a right hemicolectomy, extended to 40 cm of terminal ileum, followed by a side-to-side anastomosis (Figure 3 and Figure 4).

**Figure 5:**
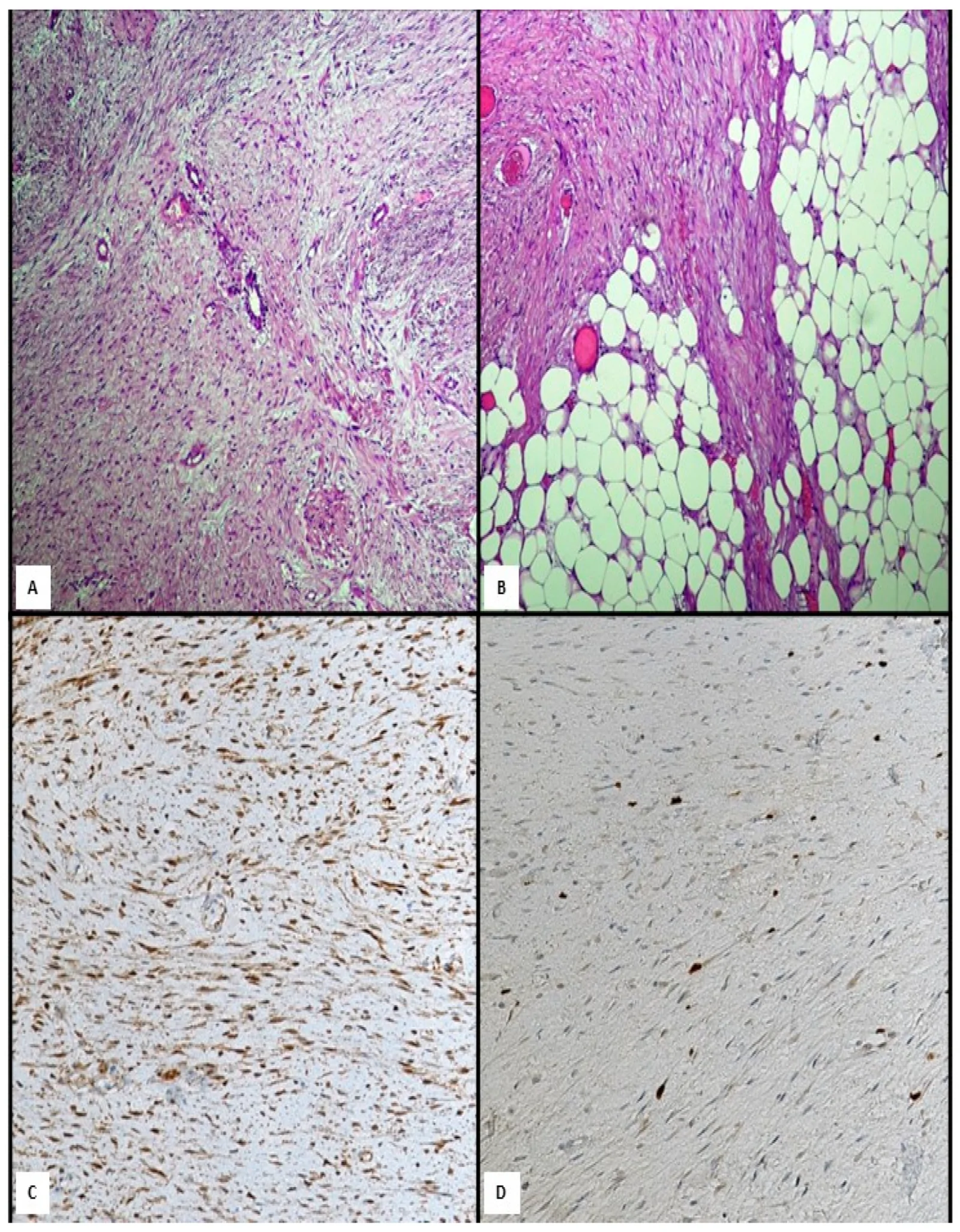
Histological analysis showed a neoplastic proliferation composed of bland spindle-shaped fibroblastic cells arranged in parallel fascicles with a regular nucleus, separated by large amounts of collagen fibers (**A**, Haematoxylin and eosin, original magnification 20×). The tumor invaded adjacent adipose tissue with infiltrating border (**B**, Haematoxylin and eosin, original magnification 20×). Immunohistochemistry showing positive nuclear β-catenin staining (**C**, Mayer’s hemalum counterstain, original magnification 20×). The tumor cells showed very low Ki-67 labeling index (**D**, Mayer’s hemalum counterstain, original magnification 20×).

Histological examination (Figure 5) of the intestinal wall revealed a spindle cell proliferation of moderate cellularity, arranged in fascicles within a variably collagenous stroma. No areas of necrosis were identified, and mitotic figures were rare, indicating a low proliferative index. Examination of 41 regional lymph nodes showed nonspecific reactive lymphadenitis, with no evidence of metastatic involvement. The appendix and surgical resection margins were free of pathological involvement. Immunohistochemical analysis demonstrated strong, diffuse nuclear expression of beta-catenin, supporting the diagnosis of desmoid-type fibromatosis, together with a weakly positive Ki-67 proliferation index, consistent with the low mitotic activity observed on hematoxylin–eosin staining. To exclude the principal differential diagnoses of an intra-abdominal spindle-cell lesion, the following additional markers were assessed and were all negative: CD117 (c-KIT) and CD34, excluding gastrointestinal stromal tumor and solitary fibrous tumor (SFT); smooth muscle actin (SMA) and desmin, excluding a smooth muscle tumor (leiomyoma/leiomyosarcoma); S100, excluding a peripheral nerve sheath tumor; and cytokeratin AE1/AE3, excluding a carcinomatous/epithelial origin. DOG1 and ALK were not performed. Together, this immunophenotype (beta-catenin strongly positive; CD117, CD34, desmin, SMA, S100 and cytokeratin AE1/AE3 negative; DOG1 and ALK not performed) was considered characteristic of desmoid-type fibromatosis and inconsistent with GIST, solitary fibrous tumor, smooth muscle tumor, peripheral nerve sheath tumor, or carcinoma.

The postoperative course was uneventful, and the patient was discharged on 5 POD. Given the recognized association between desmoid tumors and familial adenomatous polyposis, a screening colonoscopy was performed during follow-up at two months after surgery to exclude colorectal polyposis; no polyps were identified. Somatic CTNNB1 mutation analysis and formal clinical genetics counseling were not performed, as the colonoscopy findings were reassuring and there was no additional clinical suspicion of a polyposis syndrome; the absence of molecular confirmation is acknowledged as a limitation of the diagnostic work-up.

No adjuvant postoperative therapy was administered.

Postoperative surveillance follows a risk-adapted, symptom-driven strategy consistent with current guideline recommendations for desmoid-type fibromatosis, comprising clinical evaluation with cross-sectional imaging (contrast-enhanced CT or MRI) at approximately 6-month intervals for the first 2 years and annually thereafter, continued for as long as the patient remains at recurrence risk [9]. The patient is under follow-up, with no evidence of recurrence to date, after 18 months of follow-up.

**Figure 6:**
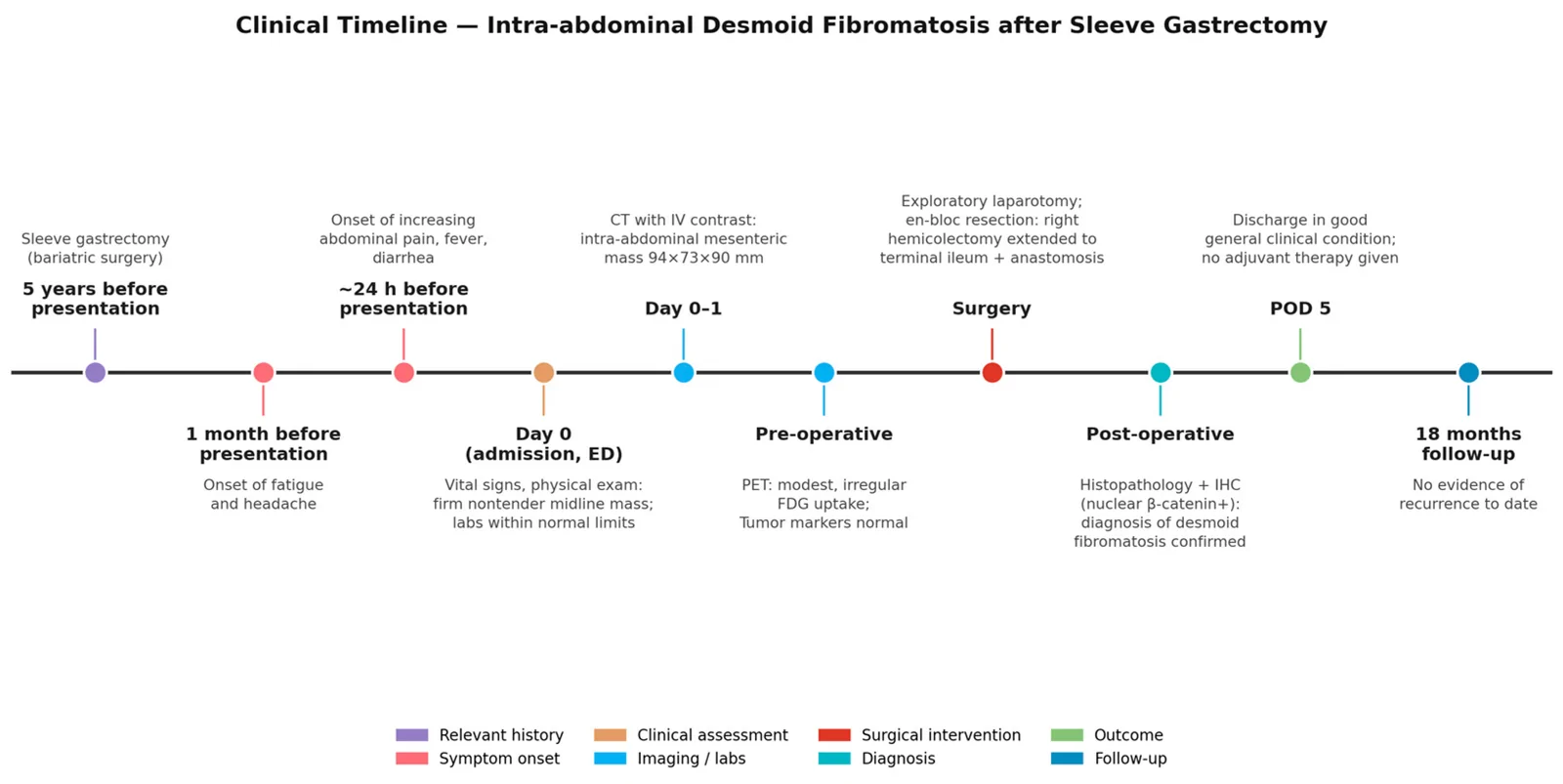
Clinical timeline of a 27-year-old man with intra-abdominal desmoid fibromatosis diagnosed 5 years after sleeve gastrectomy, from symptom onset through surgical resection, histopathological confirmation, and 18-month follow-up without recurrence.

To reconstruct the clinical course in accordance with the CARE (CAse REport) reporting guidelines, the timeline of relevant past history, symptom onset, diagnostic work-up, intervention, diagnosis confirmation, and follow-up is summarized in Table 1 and Figure 6.

## 3. Discussion

DF is a rare subtype of soft tissue neoplasm, with an annual incidence of 2 to 4 cases per million people aged 15 to 60, showing a slight predilection for women. They account for less than 1% of retroperitoneal masses, 0.03% of all neoplasms, and less than 3% of soft tissue tumors [2]. DF is classified as an intermediate-grade neoplasm that does not have metastatic or dedifferentiation potential, but exhibits aggressive local infiltrative behavior [3].

DTs usually appear in two common forms: “sporadic”, often associated with past traumatic or surgical injuries, and “genetic”, linked to Gardner syndrome (i.e., familial adenomatous polyposis). This genetic form occurs in 10–30% of cases [8].

DTs most commonly develop in the abdominal region, accounting for 37–50% of cases. They can originate either from the deep soft tissues of the abdominal wall or from intra-abdominal structures (10–20% of all cases) [2,7]. Intra-abdominal DTs most frequently arise from the small bowel mesentery, which is their primary site of occurrence. Other locations include omentum, mesocolon and colonic wall [4].

The shoulder girdle, chest wall and inguinal regions represent frequent extra-abdominal locations.

The pathogenesis of DTs remains poorly understood. Nevertheless, factors such as female gender, estrogen exposure, prior abdominal surgeries, and trauma have been identified as potential contributors to their development. These factors should alter the normal regulation of connective tissue growth, leading to tumor formation [10]. In our case, the patient presented a DT following bariatric surgery (sleeve gastrectomy).

We were interested by this observation and conducted a review of the literature to explore whether a hypothesis-generating association between bariatric surgery and DT could be identified, while recognizing that case reports alone cannot establish a true statistical correlation. We performed research on the PubMed and Embase databases up to 31st December 2025 using the following Boolean search strings. PubMed: (“desmoid tumor”[Title/Abstract] OR “desmoid fibromatosis”[Title/Abstract] OR “aggressive fibromatosis”[Title/Abstract] OR “mesenteric fibromatosis”[Title/Abstract]) AND (“bariatric surgery”[Title/Abstract] OR “sleeve gastrectomy”[Title/Abstract] OR “gastric bypass”[Title/Abstract] OR “obesity surgery”[Title/Abstract]). Embase: an equivalent Emtree-based string combining the subject headings ‘desmoid tumor’/‘aggressive fibromatosis’/‘mesenteric fibromatosis’ with ‘bariatric surgery’/‘sleeve gastrectomy’/‘gastric bypass’, using the OR/AND operators as above. No language or publication-date restriction was applied.

Inclusion criteria were (i) case reports or case series describing desmoid tumor/desmoid-type fibromatosis occurring after any bariatric or metabolic surgical procedure; (ii) human subjects; (iii) sufficient clinical detail to extract patient age, sex, type of bariatric surgery, interval to diagnosis, tumor site, treatment, and outcome; and (iv) full text available in English. Exclusion criteria were (i) desmoid tumors clearly attributable to familial adenomatous polyposis without an antecedent bariatric procedure; (ii) reviews, commentaries, or conference abstracts without extractable individual patient data; (iii) duplicate reports of the same patient; and (iv) non-English full text with no available translation.

**Figure 7:**
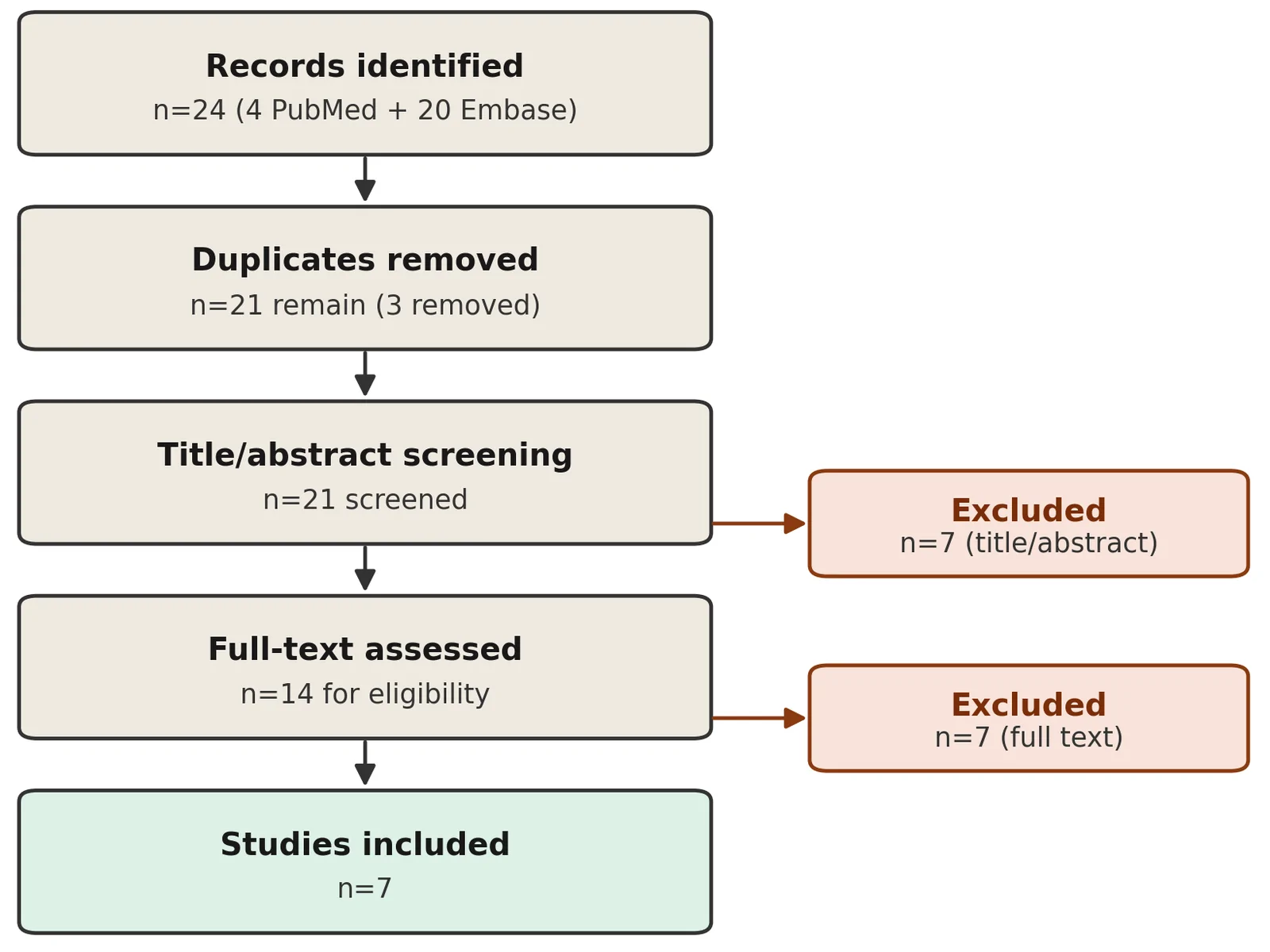
PRISMA flow diagram of the literature search and study selection process [11,12,13,14,15,16,17].

Titles and abstracts were independently screened by two authors (T.S. and F.F.); full texts of potentially eligible records were then reviewed in duplicate, and disagreements were resolved by discussion with a third author (M.I.). Of the 24 records identified through database searching (4 from PubMed and 20 from Embase), 3 duplicates were removed and 21 records were screened. At the title/abstract stage, 7 records were excluded (non-English language, n = 1; unrelated topic, n = 4; wrong tumor entity, n = 1; wrong surgical exposure, n = 1). The remaining 14 full texts were assessed for eligibility; 7 were excluded (wrong tumor entity, n = 1; unrelated topic, n = 1; insufficient extractable case data in conference-abstract or inaccessible sources, n = 3; desmoid tumor arising after gastric cancer surgery rather than bariatric surgery, n = 2), leaving 7 studies that met the inclusion criteria [11,12,13,14,15,16,17] (Figure 7). Reference lists of all eligible articles and of relevant narrative reviews were additionally hand-searched, which did not identify further eligible cases.

We found seven previously reported cases of FD after bariatric surgery, following mini-gastric bypass (n = 1), Roux-en-Y gastric bypass (n = 2), sleeve gastrectomy (n = 3), and gastric bypass (n = 1), respectively; one of these (Maalouf et al. [17]) has a less clearly defined temporal relationship to the bariatric procedure—see Table 2 footnote (summarized together with the present case in Table 2) [11,12,13,14,15,16,17].

As shown in Table 2, the eight cases (seven from the literature and the present one) were managed surgically, with no recurrence reported at last follow-up whenever follow-up was available. Most cases involved the small bowel mesentery, typically diagnosed 2–8 years after the bariatric procedure; two cases (Sedeyn et al., Aljomah et al. [15,16]) instead presented as an upper-abdominal or gastric mass adjacent to the surgical site, indicating that tumor location is not restricted to the small bowel mesentery. This descriptive pattern, drawn from a small and heterogeneously reported sample (including conference-abstract sources with limited follow-up data), is presented as a hypothesis-generating observation rather than proof of an underlying association.

On the other hand, there may be an association between DT, obesity and hormonal alterations.

In a study assessing the therapeutic efficacy of pegylated liposomal doxorubicin (PLD) [18], it was notable that 31 of the 61 patients had a documented history of psychiatric disorders, primarily depression and anxiety. Furthermore, among the 41 female patients, six reported either a personal or familial history of polycystic ovary syndrome (PCOS). Additionally, 15 patients were obese and 4 had hypothyroidism.

Several biological mechanisms have been proposed to link metabolic and hormonal dysregulation to desmoid tumor pathogenesis. Obesity-associated chronic low-grade inflammation and increased peripheral aromatization of androgens to estrogens within adipose tissue raise local and systemic estrogen exposure, which has long been implicated in DF growth, consistent with its higher incidence in women of reproductive age. Hyperinsulinemia and insulin resistance, common in obesity, PCOS, and post-bariatric metabolic states, may further promote fibroblast proliferation through insulin/IGF-1 signaling, which intersects with the Wnt/beta-catenin pathway that is constitutively activated in DF through somatic CTNNB1 mutations. This convergence between metabolic/hormonal signaling and Wnt/beta-catenin activation offers a biologically plausible, though still unproven, mechanistic link between conditions such as obesity, PCOS, and hyperinsulinemia and the development of DF. Hence, we suppose that hormonal and metabolic imbalance, together with surgical trauma, may activate metabolic processes underlying the development of DT, a hypothesis that warrants dedicated mechanistic and epidemiological investigation.

The symptoms can vary according to the tumor’s location. The absence of specific symptoms makes diagnosis challenging. Most patients report an asymptomatic abdominal mass, although some may experience abdominal discomfort or obstructive symptoms. Pain does not correlate with tumor size. Mesenteric DTs may lead to acute abdominal pain, hemorrhage, or peritonitis secondary to bowel perforation [5].

Diagnosing DF can be challenging, as no definitive test is available. The diagnostic process typically involves multiple steps, including obtaining the patient’s medical history, conducting a physical examination, utilizing diagnostic imaging, analyzing a tumor biopsy and eventually, performing genetic testing.

According to National Comprehensive Cancer Network (NCCN) guidelines [9], CT is the preferred imaging method for diagnosing intra-abdominal DF. It is particularly effective for identifying complications such as intestinal obstruction, perforation, mesenteric ischemia, fistulization, and abscess formation. Magnetic Resonance Imaging (MRI), meanwhile, serves as a valuable complementary tool, offering the additional advantage of better detecting small necrotic areas within the lesion.

Imaging plays a crucial role in preoperative assessment by providing valuable insights into vascular involvement, the extent of invasion and the size of the mass.

Abdominal masses can occur in a variety of pathological conditions including infectious or inflammatory processes, lymphomas, fibrosarcomas, GISTs, and mesenteric metastases. Definitive diagnosis relies on histopathological evaluation, with immunohistochemical analysis typically revealing positivity for smooth muscle actin and nuclear beta-catenin [19].

Immunohistochemistry plays a central role in differentiating DF from other spindle-cell lesions of the abdominal cavity. Nuclear beta-catenin expression, resulting from constitutive activation of the Wnt/beta-catenin pathway via somatic CTNNB1 mutations or, less commonly, germline APC mutations, is detected in the majority of cases and represents the diagnostic hallmark, although a negative result does not exclude DF [9,18]. Smooth muscle actin is frequently expressed, reflecting the myofibroblastic differentiation of tumor cells, whereas desmin is typically negative or only focally positive; this pattern helps distinguish DF from leiomyoma and leiomyosarcoma, which usually show diffuse and strong desmin and h-caldesmon positivity [20]. CD117 (c-KIT) and DOG1 are essential to exclude GIST, the most frequent differential diagnosis for an intra-abdominal spindle-cell mass, and are consistently negative in DF. CD34 is also negative, distinguishing DF from solitary fibrous tumor and from CD34-positive GISTs [20]. ALK immunostaining—positive in 50–70% of inflammatory myofibroblastic tumors due to ALK gene rearrangement—is negative in DF, while S100 negativity excludes a peripheral nerve sheath tumor [21]. A low Ki-67 proliferation index further supports the intermediate, locally aggressive but non-metastasizing biological behavior of DF [9].

The spectrum of desmoid tumors is broad, including different histological patterns such as myxoid, nodular fasciitis-like, hypocellular and keloidal-like patterns. On immunohistochemistry, the hallmark feature of the tumor is the nuclear accumulation of beta-catenin. Mutational analysis (involving mutations of the CTNNB1 gene and APC gene) might be required in challenging cases, especially in beta-catenin negative tumors and in hereditary cases [22,23].

Management of DF requires a multidisciplinary approach. Although surgical resection remains the standard treatment for symptomatic cases, recent research has explored alternative strategies, especially for asymptomatic patients. These approaches include active surveillance, radiotherapy (RT), and systemic medical therapy. Options for uncomplicated, unresectable, or recurrent DF include nonsteroidal anti-inflammatory drugs (NSAIDs), antioestrogens (e.g., tamoxifen), and cytotoxic chemotherapy (e.g., low-dose methotrexate with vinblastine, or anthracycline-based regimens), in addition to pharmacological treatment such as estrogen receptor blockers and tyrosine kinase inhibitors (e.g., sorafenib) [24,25].

Some cases of DF have been reported to undergo spontaneous regression [1].

Surgical management is determined by key factors such as tumor location, size, and extent of local invasion. Although R0 resection remains the standard of care, resections with microscopically positive margins are considered acceptable. This is supported by evidence indicating that recurrence rates are not significantly impacted by the presence of microscopically positive margins [26].

Following surgical resection, diligent and close follow-up is imperative due to the notable recurrence rates, particularly in intra-abdominal cases where rates range from 15% to 30% [27]. However, no standardized surveillance protocol has been established.

RT represents a viable therapeutic option for management. Its application is primarily reserved for cases where surgical excision is not feasible or as an adjunctive therapy following resections with positive margins (non-R0 resections). The role of RT continues to be refined in clinical practice, particularly in complex cases [28].

Postoperative RT has been identified as an independent favorable prognostic factor for local control and progression-free survival. However, this is observed exclusively for extra-abdominal and abdominal wall desmoids [29]. According to the current literature, intra-abdominal DTs that undergo R0 resection do not require adjuvant radiation.

Prognostic factors associated with poor outcomes include young age (under 37 years), tumor size (greater than 7 cm), extra-abdominal tumor location, and the quality of surgical resection [30].

In summary, the observations presented in our study, together with the seven cases previously reported in the literature, raise a hypothesis-generating question about a possible link between bariatric surgery and the development of desmoid tumors, rather than establishing a true correlation. Given that bariatric surgery is a common procedure while desmoid fibromatosis remains an exceedingly rare neoplasm, and that only eight cases (including ours) have been identified worldwide, the current evidence is insufficient to support a genuine association and should be interpreted with caution. To our knowledge, this review is the first to raise this hypothesis; although the total number of cases remains limited, we believe the observation warrants further investigation rather than representing a confirmed clinically relevant signal. Hormonal and metabolic disturbances, such as insulin resistance and hyperinsulinemia—and, in women, hyperandrogenism associated with PCOS—appear to contribute to a biological microenvironment conducive to abnormal cell proliferation. These mechanisms are well established in the context of hormone-dependent neoplasms; however, their possible involvement in the pathogenesis of desmoid tumors remains poorly investigated, and no causal link has been established. To date, comprehensive endocrine evaluation is not routinely integrated into oncological clinical protocols. This gap may lead to underdiagnosis or an incomplete assessment of neoplastic risk in patients affected by obesity and PCOS.

Taken together, the choices made in this case—imaging tailored to the patient’s specific anatomical presentation, an intraoperative surgical strategy adapted to the tumor’s relationship with the terminal ileum, and a risk-adapted, symptom-aware follow-up plan—exemplify how DF management is necessarily individualized rather than protocol-driven. We believe this case adds to the growing body of evidence supporting personalized diagnostic and therapeutic pathways for rare, heterogeneous neoplasms such as DF, particularly in atypical clinical contexts such as prior bariatric procedures. We wish to reiterate, however, that with only eight cases reported worldwide to date, no causal or even statistically supported relationship between bariatric surgery and desmoid fibromatosis can be inferred from the available evidence; the observation remains strictly hypothesis-generating and should be interpreted with corresponding caution by readers and future investigators alike.

## 4. Conclusions

Intra-abdominal DF is a rare, locally aggressive condition that, despite being histologically benign, requires an individualized, multidisciplinary diagnostic and therapeutic strategy, as illustrated by our patient. Although surgical emergencies are uncommon, they can occur, and R0 resection remains the treatment of choice. This case adds to the limited literature describing DF after bariatric surgery and reinforces the need for larger, multicenter studies to clarify this association, standardize postoperative surveillance, and better define the role of metabolic and hormonal factors in DF pathogenesis. Given the small number of cases reported to date, this association remains speculative and should not be interpreted as evidence of causation.

## Figures and Tables

**Table 1:** Timeline of clinical events, diagnostic evaluations, interventions, and outcomes, reconstructed according to the CARE guidelines and referenced to their location in the manuscript.

Timepoint	CARE Category	Clinical Event/Finding	Source in Text
–5 years	Relevant past history	Sleeve gastrectomy (bariatric surgery) performed; also remote tonsillectomy. No history of drug abuse, allergies, or relevant family history.	Materials and Methods
–1 month	Symptom onset	Onset of fatigue and headache, persisting for 1 month prior to admission.	Materials and Methods
~24 h before presentation	Symptom onset/worsening	Increasing lower abdominal pain, fever, and diarrhea for approximately 24 h.	Materials and Methods
Day 0 (admission)	Clinical presentation	Presentation to the Emergency Department. Vital signs: T 37.8 °C, BP 120/80 mmHg, HR 110 bpm, RR 16/min. Laboratory tests within normal limits. Abdominal examination: firm, nontender midline mass.	Materials and Methods
Day 0–1	Diagnostic imaging	CT with IV contrast: intra-abdominal mesentery-based mass, 94 × 73 × 90 mm, inseparable from ileal loops, displacing right colic artery branches; multiple lymphadenopathies (≤15 mm); no thoracic/abdominal organ involvement.	Materials and Methods; Figure 1
Pre-operative	Diagnostic imaging	PET: modest, irregular FDG uptake at the mass; low and inhomogeneous pelvic metabolic activity related to an abundant fluid component.	Materials and Methods; Figure 2
Pre-operative	Laboratory work-up	Tumor markers within normal range: CEA 0.85 ng/mL, AFP 1.20 ng/mL, CA 19-9 2.29 IU/mL, CA 125 2.68 U/mL, CA 15-3 14.70 IU/mL, CA 50 1.70 U/mL.	Materials and Methods
Day of surgery (POD 0; day 5 after admission)	Therapeutic intervention	Exploratory laparotomy; en-bloc resection of the mass with right hemicolectomy extended to 40 cm of terminal ileum; side-to-side anastomosis.	Materials and Methods; Figure 3 and Figure 4
Post-operative	Diagnostic confirmation	Histopathology: spindle-cell proliferation in fascicles, low proliferative index, clear margins; 41 regional lymph nodes, all reactive (no metastatic involvement). Immunohistochemistry: nuclear β-catenin strongly positive, weakly positive Ki-67; CD117, CD34, desmin, SMA, S100 and CK AE1/AE3 negative → diagnosis of desmoid-type fibromatosis confirmed.	Materials and Methods; Figure 5
POD 5	Clinical outcome	Uneventful postoperative course; discharged in good general clinical condition. No adjuvant postoperative therapy administered.	Materials and Methods
18 months	Follow-up	Patient remains under follow-up, with no evidence of recurrence to date.	Materials and Methods; Conclusions

CARE: CAse REport guidelines; BP: Blood Pressure; HR: Heart Rate; CT: Computed Tomography; IV: Intravenous contrast medium; PET: Positron Emission Tomography; FDG: Fluorodeoxyglucose; CEA: Carcinoembryonic Antigen; AFP: Alpha-Fetoprotein; CA: Cancer Antigen; POD: Postoperative Day; CD: Cluster of Differentiation; SMA: Smooth Muscle Actin; CK AE: Cytokeratin AE1/AE3 .

**Table 2:** Summary of previously reported desmoid tumor/desmoid-type fibromatosis cases following bariatric surgery, and the present case (highlighted).

Study	Age/Sex	Bariatric Surgery	Interval to Diagnosis	Tumor Site	Treatment	Outcome
Mahnashi et al. [11] (2020)	48/M	Mini-gastric bypass (OAGB)	3 years	Gastrojejunal anastomosis (mesenteric supply)	En-bloc resection; Roux-en-Y reconstruction	No recurrence reported
Sierra-Davidson et al. [12] (2020)	27/F	Roux-en-Y gastric bypass	2 years	Small bowel mesentery (Roux limb/jejunojejunostomy)	En-bloc resection with Roux limb; RYGB reconstruction	Uneventful short-term recovery; long-term recurrence not reported
Medas et al. [13] (2023)	26/M	Sleeve gastrectomy	3 years	Small bowel mesentery	R0 resection + segmental enterectomy	No recurrence at 4-year follow-up
Shafi et al. [14] (2022)	74/F	Gastric bypass	8 years	Mesentery of small intestine	Surgical resection alone (no systemic therapy)	No recurrence at 18 months
Sedeyn et al. [15] (2015) *	44/NS	Sleeve gastrectomy	~2 years	Left upper abdomen (mesenteric/retroperitoneal, compressing stomach; displacing bowel, pancreas, kidney, spleen)	En-bloc resection, distal pancreatectomy, splenectomy, partial gastrectomy with Roux-en-Y reconstruction	Not reported (abstract only)
Aljomah et al. [16] (2025) *	36/M	Sleeve gastrectomy	2 years	Gastric mass (7.4 cm) at staple line, inseparable from stomach and transverse colon	Diagnostic laparoscopy, subtotal gastrectomy with en-bloc resection of transverse colon	Uneventful postoperative course (recurrence not reported; abstract only)
Maalouf et al. [17] (2024) †	43/F	Roux-en-Y gastric bypass † (after initial SFT resection)	~2 years	Mesentery, 80 cm from ligament of Treitz, in contact with small bowel	En-bloc resection + partial enterectomy, end-to-end anastomosis	Not reported beyond immediate postoperative course
Present case	27/M	Sleeve gastrectomy	5 years	Small bowel mesentery (terminal ileum)	Right hemicolectomy extended to terminal ileum + anastomosis	No recurrence at 18 months

* Sedeyn et al. [15] and Aljomah et al. [16] are conference-abstract reports; granular follow-up/recurrence data were not available. † Maalouf et al. [17]: the patient’s Roux-en-Y gastric bypass predated a first tumor (a solitary fibrous tumor (SFT) initially mistaken for GIST); the desmoid tumor listed here was a second, histologically distinct mesenteric lesion detected on interval imaging approximately 2 years after resection of the SFT—the exact interval from the bariatric surgery itself to the desmoid diagnosis is not stated in the source. RYGB: Roux-en-Y Gastric Bypass.

## Data Availability

The original contributions presented in this study are included in the article. Further inquiries can be directed to the corresponding authors.
